# Supplementary material for: A multistudy analysis reveals that evoked pain intensity representation is distributed across brain systems
Source: PLoS Biol. 2022 May 2;20(5):e3001620. doi: 10.1371/journal.pbio.3001620 (PMC9098029; doi:10.1371/journal.pbio.3001620)
Supplement: S1 Text — (DOCX) [file pbio.3001620.s001.docx]

**SUPPLEMENTAL RESULTS**

*Models capture pain specific representation: studies V1 and V4*

Our primary analysis of pain specificity estimates pain intensity from a single mean contrast map for each participant across 18 studies with putatively homomorphic evoked responses (Fig 8). Meanwhile, our analysis of pain sensitivity measures correlations of predicted and observed pain across multiple single trial maps spanning a range of intensities for each participant (Fig 5). We used multimodal data from studies V1 and V4 to compare approaches using mean contrast maps with those using single trial maps. We found they yield congruent measures.

In study V1 participants rated perceived warmth of noxious heat stimuli (>40°C) which were subjectively not painful, while in study V4 participants also rated the aversiveness of unpleasant sounds from the international affective digital sounds database. On average our models tracked both the aversiveness of sound (mean zr = 0.22, p = 0.0012, t_87_ = 3.34) and warmth of nonpainful noxious heat (mean r = 0.48, p = 3.1e-4, t_31_ = 4.1, mixed model, random participants; S3 Fig). Post-hoc tests identified several of our models in particular which decoded either sound (pain pathways p = 5.6e-4) or warmth (right salience/ventral attention A (fRSN) p = 9.8e-4, frontoparietal (cRSN) p = 0.0025, pain pathways p = 0.0010 and neurosynth p = 5.4e-4, t-tests, Sidak corrected for 12 comparisons), while remaining models were mostly ambiguous (not significant, but BF in favor of null prediction < 10 in all cases, except the a24pr [best region] model which did not predict sound, BF = 10.4). However, models tracked pain more accurately than nonpainful noxious heat (z-Fisher r = 0.22 higher on average for painful heat, t_341_ = 2.5, p = 0.01, mixed model, random participant) or aversive sound (z-Fisher r = 0.12 higher for pain than sound, t_948.6_ = 2.0, p = 0.04). At least one model in particular was more sensitive to pain than sound (neurosynth p = 0.001, Sidak corrected for 12 comparisons) and painful rather than nonpainful heat (region a24pr p = 0.006). Importantly, this advantage is due to the pattern of activation, not net amplitude differences across modalities, because all brain maps in this study were normalized to have unit amplitude during preprocessing. These results are suggestive of some (but certainly not exclusive) pain specificity, albeit their inferential abilities are limited, since model scores were each obtained in only one single study, and there is considerable variability in performances of models from different studies (Fig 5B).

Scores on these same models were also estimated from individual participant mean stimulus evoked contrast maps. Mean scores were categorically larger for pain than non-pain conditions (S3 Fig, right), and magnitude of response tracked specificity of single trial scores. Unlike scores on single trial evoked responses, all models showed significantly greater specificity for pain on mean contrast maps. This advantage is likely attributable to improved signal to noise ratio obtained by averaging over a greater number of stimulus events. This congruence between single trial and mean contrast map results allows us to test specificity across a broader class of conditions in our main validation analysis, including many where single trial evoked responses are unavailable.

#### 
