# Supplementary material for: A multistudy analysis reveals that evoked pain intensity representation is distributed across brain systems
Source: PLoS Biol. 2022 May 2;20(5):e3001620. doi: 10.1371/journal.pbio.3001620 (PMC9098029; doi:10.1371/journal.pbio.3001620)
Supplement: S2 Text — (DOCX) [file pbio.3001620.s002.docx]

**SUPPLEMENTAL METHODS**

**Behavioral data analysis: Partial r^2^ computation**

Partial coefficient of determination (r^2^) quantifies variance explained by factors in multiple regression models. Formal definitions vary, and the most common are marginal, quantifying the amount of additional variance explained by the inclusion or omission of a variable from a model, e.g. [[1]](https://paperpile.com/c/PfkOzA/YkQza), however, when variables have any collinearities they will show some overlap in the outcome variance they can explain which marginal r^2^ calculations omit. We instead adopted the method described by [[2]](https://paperpile.com/c/PfkOzA/ujxWb) which splits the difference for collinear independent variables. We took the covariance of the independent variable with predicted pain report (a univariate analysis) and scaled it by the standardized regression coefficient of the multivariate model of pain report just described. Computed this way, the partial r^2^ sum to 1, facilitating their naive interpretation as “percent variance explained”.

**Defining model spaces and inputs**

*Elementary regions map*

486 regions of interest subdivided the brain for anatomical characterization of sources of predictive information. These regions comprehensively cover brain gray matter from the brainstem through the forebrain, and are based on parcels and subcortical nuclei defined in previous published studies. We obtained 360 cortical regions (180 areas x 2 hemispheres) from the Human Connectome Project’s cortical parcellation, which delineates areas according to neuroimaging derived anatomical and multitask functional criteria [[3]](https://paperpile.com/c/PfkOzA/5uMdP), transformed to volumetric MNI space. T1 and T2 based anatomical criteria delineated striatum, ventral tegmental area, substantia nigra, bed nucleus of the stria terminalis, pallidum, mammillary nuclei and the habenula (25 regions) based on [[4]](https://paperpile.com/c/PfkOzA/4kxQr). T1 based anatomical criteria delineated the cerebellar lobules, vermis and deep nuclei (denate, interposed, fastigial; 34 regions total) [[5]](https://paperpile.com/c/PfkOzA/hUzgj). T1 derived tissue boundaries and white matter tractography delineated 16 thalamic nuclei and the hypothalamus [[6]](https://paperpile.com/c/PfkOzA/Z2AX5). A variety of nucleus specific functional and anatomical criteria defined monoaminergic brainstem nuclei, the rostroventral medulla, parabrachial nuclei, trigeminal nuclei, nucleus ambiguus, nucleus tractus solitarius, tegmental nuclei, and some subdivisions thereof (24 areas) [[7–16]](https://paperpile.com/c/PfkOzA/tu51L+W9YMz+nxFnW+WbyZt+aRlNu+3yBwg+LhD9i+ucfKQ+916xP+k5CYJ). Functional homogeneity criteria from a graph theoretic analysis of functional connectivity delineated remaining gross divisions of the brainstem (17 areas) [[12]](https://paperpile.com/c/PfkOzA/3yBwg). Finally, anatomical criteria adapted from post-mortem studies and included in the SPM Anatomy Toolbox subdivided hippocampal formation into CA1, CA2, CA3, dentate gyrus and subiculum, as well as gross subdivisions of the amygdala (superficial, latero-basal, centro-medial) (9 regions) [[17,18]](https://paperpile.com/c/PfkOzA/5Hr7w+xeG15).

*Network parcellation maps*

We used two different resting state network parcellations of the cortex as source regions for pain intensity prediction. One involved 7 networks (coarse resting state networks, cRSN), and the other was a finer parcellation of 17 networks (fine resting state networks, fRSN). cRSN regions were bilateral, while the fRSN regions were largely unilateral (32 regions total), and were derived in the same study sample [[19]](https://paperpile.com/c/PfkOzA/yYBlQ).

*Pain Pathways Map*

We manually created a map by combining areas from the elementary regions map (above) by selecting regions related to pain in previous literature [[20,21]](https://paperpile.com/c/PfkOzA/qYs7z+J1ZPJ) and regions that play important roles in non-human pain studies. The areas included were ventral posterolateral, intralaminar and mediodorsal thalamic nuclei, hypothalamus, parabrachial nuclei, periaqueductal gray matter (PAG), rostroventral medulla, amygdala, dorsal posterior, middle and anterior insula, primary and secondary somatosensory cortices, and anterior medial cingulate cortex/medial prefrontal cortex (PFC). Regions were selected based on the prior knowledge of the field (by T.W.D.). The map and labels are available online in the Cognitive and Affective Neuroscience Laboratory Github repository at <https://github.com/canlab/Neuroimaging_Pattern_Masks/>.

*Neurosynth.org empirical occurrence map*

Following the approach of [[22]](https://paperpile.com/c/PfkOzA/V7IID)⁠, we defined an empirical map delineating regions which occur in neuroimaging studies of pain by searching neurosynth.org [[23]](https://paperpile.com/c/PfkOzA/AKPet) for “pain”, and thresholded the “association test” map (a quas-“reverse inference” map derived from frequentist test results) at the false discovery rate (FDR) q < 0.05.

**Multivariate Pattern Analysis (MVPA): Bayesian Optimization**

Bayesian hyperparameter optimization is a sophisticated optimization approach that uses Gaussian process regression to combine limited sampling with smoothness priors instead of exhaustively sampling all possible hyperparameter combinations. The posterior distribution over loss functions (MSE in this case) is estimated after each sampling and used to select the next point to sample, stopping after 30 iterations (our implementation’s default stop condition). There are two major advantages to this approach to PCR dimension selection over other methods like grid sampling. First, cross validation is subject to sampling variance (different fold slicing produces slightly different results). Gaussian process regression models this variance as noise and takes this noise into account when selecting global optima. Second, estimating a loss function after each sampling iteration allows for intelligent selection of the next best point to sample (for instance sampling in the domain which maximally improves precision of posterior loss function estimates), thus accelerating convergence on a solution in what might be complex solution spaces. We used the algorithm as implemented in the BayesianOptimization toolbox in Matlab [[24]](https://paperpile.com/c/PfkOzA/iKPy4)⁠.

**MVPA Bootstrap Tests**

This study used bootstrap resampling to generate brain maps representative of the study population (Fig 4).

Bootstrap resampling identified statistically significant voxels (uncorrected, bootstrapped 95% CI does not include 0) for multisystem predictive models (Neurosynth and pain pathways) and the full brain predictive model. Additionally, we used bootstrap resampling to identify significant voxels for each region, cRSN and fRSN selected by any cross validation fold. Bootstrap corrected predictive maps are fit to the entire dataset (balanced across studies and participants but not resampled), but thresholded to only show voxels that are statistically significant under the bootstrap analysis.

For any bootstrapped analysis it is problematic to identify the PCR hyperparameters, namely the dimensionality of the underlying PCA. We wished to identify the relationship between PCA dimensionality and the expected generalization performance of the obtained PCR models, a procedure which generally relies on some form of cross validation (see Methods subsection Multivariate Pattern Analysis [MVPA]: General Method Description). However, cross validation is incompatible with bootstrapping since redundancy of resampled observations in both training and test splits violates independence assumptions. This confounds hyperparameter selection through inner cross validation loops. A common approach is to estimate model error as a function of the hyperparameters in the original, non bootstrapped sample, and then reuse that point estimate of the optimum dimension for PCR in the bootstrapped samples. However, there is some uncertainty in the estimate of the optimal hyperparameter which our Bayesian hyperparameter optimization scheme allowed us to readily integrate into bootstrapped analysis.

Bayesian optimization of hyperparameters estimates a loss function of the hyperparameters (e.g. *error = f(dimensions)*) using Gaussian process regression, which entails computing a posterior probability distribution over loss functions. This posterior models uncertainty in the model estimate. We computed this posterior using the full dataset, and drew an instance *f** from the posterior distribution over *f*, which varied according to the gaussian process model’s estimate of uncertainty in *f*, computed the global minima of *f**, and used that as the hyperparameter for the bootstrapped PCR. This method is novel to our knowledge, results in greater MVPA map variance across bootstrap iterations, and more conservative bootstrap correction than typical in other MVPA studies.

**Bayesian hypothesis tests**

We assessed support for null hypotheses using Bayes factors (Fig 5, 7 & 8). Bayes factor tests are goodness-of-fit ratios that compare how well one model specification vs. another accounts for data while taking into account all possible model parameter values and their likelihoods. In all cases where we tested support for the null we first began with a frequentist test which failed to reject the null, i.e. an effect of interest lacked statistical significance (p > 0.05). This constituted the ‘alternative model’. Null models tested were all nested instances of the ‘alternative’ model lacking this parameter which fell short of significance in frequentist testing. Non-significant parameters of interest in alternative models often have mixed sources of error (e.g. random noise and participant or study related random effects). In such cases we removed all factors associated with parameters of interest, including associated random effects, in null models. Following [[25]](https://paperpile.com/c/PfkOzA/0qOTf), this study considers a Bayes factor greater than 10 in favor of the null “strong” enough to confirm the null.

We implemented Bayesian models using one of two methods. In most cases we used Markov chain Monte Carlo (MCMC) sampling algorithms specified in the STAN programming language [[26]](https://paperpile.com/c/PfkOzA/AyMr0). The rstanarm package efficiently and reproducibly converts linear mixed effects model specifications used for frequentist testing into STAN source code for null hypothesis confirmation [[27]](https://paperpile.com/c/PfkOzA/QSGy8). rstanarm by default uses a variance scaled normal(0, 2.5*σ_x_/σ_y_) prior on regression coefficients. This scale implies larger effect sizes than we believe are plausible here and we instead used normal(0, σ_x_/σ_y_). This strategy mirrors that of [[28]](https://paperpile.com/c/PfkOzA/GFkYc) who adopt a scale 1 prior on effect sizes to reflect a prior belief that effect sizes will be moderate, in our case somewhat decreases support for the null, and creates instances where we can neither reject nor accept the null, meaning it encourages more conservative claims. We subsequently performed Bayes factor tests using bridge sampling [[29]](https://paperpile.com/c/PfkOzA/9XKMG). We evaluated four MCMC chains with a burn in of 5000 samples and subsequent drawing of 5000 samples each, and visually confirmed chain convergence by inspecting parameter trace plots and by confirming unitary Rhat statistics. Additionally, to confirm convergence between frequentist and bayesian model specifications we compared maximum a posteriori (MAP) parameter estimates in null and alternative models with estimates obtained using restricted expectation maximum likelihood (REML) as implemented by the frequentist mixed effects modeling LME4 R package [[30]](https://paperpile.com/c/PfkOzA/hIMza). This ensured models used for null hypothesis confirmation primarily reflected the data rather than Bayesian model priors.

The second method we used exclusively for one sample t-tests and relied on the BayesFactor R package [[31]](https://paperpile.com/c/PfkOzA/Y3el9), since the t-test’s null model has no coefficients (only an error variance parameter) and cannot be specified in rstanarm. To best approximate our MCMC model’s priors we used BayesFactor with a prior scale parameter of 1 on the effect size rather than the default 0.707. Although BayesFactor and rstanarm differ slightly in their prior specifications, tests which can be evaluated in both packages (e.g. Bayes factor of a paired t-test) gave convergent estimates with our chosen prior parameterizations (but not with the default parameterizations).

**Mediation analysis**

Mediation analyses explored MVPA derived models and facilitated their interpretation (Results: Mediation analyses; Fig 6). We used a pair of ordinary least squares (OLS) regression models to estimate regression coefficients. First we regressed an MVPA model prediction (the mediator) on stimulus intensity and psychological manipulations (independent variables, model a) and second we regressed pain report (the dependent variable) on the mediator and independent variables (model b). We refer to the coefficient relating the mediator to the independent variable as *α*, the coefficient relating the mediator to the dependent variable as *β*. The coefficients’ product (*α***β*) estimates the mediation effect. Because resultant distributions are often highly skewed, we used bias-corrected bootstrap sampling to estimate confidence intervals of this product [[32]](https://paperpile.com/c/PfkOzA/rylYv) using 5000 resamplings of participants, at the two-tailed alpha 0.05 level. This implementation is identical to that used by Mplus 7.11, except we resampled participants (i.e. sets of observations) instead of individual observations in accordance with independence assumptions underlying bootstrap tests. The resulting estimates are slightly more conservative than equivalent parametric estimates. We also modeled participant fixed effects (one constant term per participant), but because these effects do not vary across bootstrap iterations (bootstrapping is performed between not within participants), bootstrap distributions for these effects were not computed. Instead, we orthogonalized dependent and independent variables and mediators with respect to participant fixed effects (one constant term per participant) *a priori* before estimation of all path coefficients. Thus, all mediation models reflect mediation of within-participant pain variance.

We selected independent variables for mediation models in two steps. First, we considered sensitization and habituation effects as well as all experimental manipulations in a multiple regression analysis on pain report. Second, backwards stepwise regression iteratively removed variables that did not significantly predict pain report. We evaluated all remaining variables for mediation. MVPA model predictions were used as mediators, but were not included in the variable selection process just described.

An example of our path models can be succinctly expressed in Wilkinson notation [[33]](https://paperpile.com/c/PfkOzA/lVWIU) (common to linear model specifications in Matlab and R), bearing in mind that participant intercepts are independently estimated as fixed effects here, as follows,

model a:

brain_MVPA_prediction ~ temperature + expectation_cue + sensitization + social_cue + percieved_control + (1 | participant)

model b:

pain_report ~ brain_MVPA_prediction + temperature + expectation_cue + sensitization + social_cue + percieved_control + (1 | participant)

In this example, temperature, expectation cues, sensitization/habituation effects (note that sensitization also encompases habituation since the two only differ in sign), social cues and perceived control would all show statistically significant prediction of pain report in a multiple regression.

Like in the behavioral analysis (see “Behavioral data analysis”, above), we quantified mediation variance explained using the method of [[2]](https://paperpile.com/c/PfkOzA/ujxWb). Whereas in the behavioral analysis we used standardized regression coefficients to scale the covariance of predicted outcome with independent variables, here we scaled by the standardized indirect path coefficients (i.e. product of standardized *α* and standardized *β*, the coefficients of the indirect paths from models a and b, respectively). This value is equivalent to the marginal reduction in variance explained by mediated independent variables due to inclusion of the mediator (brain prediction) in the path b model, i.e. the reduction in variance explained by the corresponding direct effect on pain report.

##### **REFERENCES**

1. [Anderson-Sprecher R. Model comparisons and R 2. Am Stat. 1994;48: 113–117.](http://paperpile.com/b/PfkOzA/YkQza)

2. [Scherrer B. Biostatistique. Université du Québec à Montréal.; 1984.](http://paperpile.com/b/PfkOzA/ujxWb)

3. [Glasser MF, Coalson TS, Robinson EC, Hacker CD, Harwell J, Yacoub E, et al. A multi-modal parcellation of human cerebral cortex. Nature. 2016;536: 171–178.](http://paperpile.com/b/PfkOzA/5uMdP)

4. [Pauli WM, O’Reilly RC, Yarkoni T, Wager TD. Regional specialization within the human striatum for diverse psychological functions. Proc Natl Acad Sci U S A. 2016;113: 1907–1912.](http://paperpile.com/b/PfkOzA/4kxQr)

5. [Diedrichsen J, Balsters JH, Flavell J, Cussans E, Ramnani N. A probabilistic MR atlas of the human cerebellum. Neuroimage. 2009;46: 39–46.](http://paperpile.com/b/PfkOzA/hUzgj)

6. [Krauth A, Blanc R, Poveda A, Jeanmonod D, Morel A, Székely G. A mean three-dimensional atlas of the human thalamus: generation from multiple histological data. Neuroimage. 2010;49: 2053–2062.](http://paperpile.com/b/PfkOzA/Z2AX5)

7. [Zambreanu L, Wise RG, Brooks JCW, Iannetti GD, Tracey I. A role for the brainstem in central sensitisation in humans. Evidence from functional magnetic resonance imaging. Pain. 2005;114: 397–407.](http://paperpile.com/b/PfkOzA/tu51L)

8. [Fairhurst M, Wiech K, Dunckley P, Tracey I. Anticipatory brainstem activity predicts neural processing of pain in humans. Pain. 2007;128: 101–110.](http://paperpile.com/b/PfkOzA/W9YMz)

9. [Nash PG, Macefield VG, Klineberg IJ, Murray GM, Henderson LA. Differential activation of the human trigeminal nuclear complex by noxious and non-noxious orofacial stimulation. Hum Brain Mapp. 2009;30: 3772–3782.](http://paperpile.com/b/PfkOzA/nxFnW)

10. [Sclocco R, Beissner F, Desbordes G, Polimeni JR, Wald LL, Kettner NW, et al. Neuroimaging brainstem circuitry supporting cardiovagal response to pain: a combined heart rate variability/ultrahigh-field (7 T) functional magnetic resonance imaging study. Philosophical Transactions of the Royal Society A: Mathematical, Physical and Engineering Sciences. 2016;374: 20150189.](http://paperpile.com/b/PfkOzA/WbyZt)

11. [Pauli WM, Nili AN, Tyszka JM. A high-resolution probabilistic in vivo atlas of human subcortical brain nuclei. Sci Data. 2018;5: 180063.](http://paperpile.com/b/PfkOzA/aRlNu)

12. [Shen X, Tokoglu F, Papademetris X, Constable RT. Groupwise whole-brain parcellation from resting-state fMRI data for network node identification. Neuroimage. 2013;82: 403–415.](http://paperpile.com/b/PfkOzA/3yBwg)

13. [Keuken MC, Bazin P-L, Crown L, Hootsmans J, Laufer A, Müller-Axt C, et al. Quantifying inter-individual anatomical variability in the subcortex using 7 T structural MRI. Neuroimage. 2014;94: 40–46.](http://paperpile.com/b/PfkOzA/LhD9i)

14. [Keren NI, Lozar CT, Harris KC, Morgan PS, Eckert MA. In vivo mapping of the human locus coeruleus. Neuroimage. 2009;47: 1261–1267.](http://paperpile.com/b/PfkOzA/ucfKQ)

15. [Beliveau V, Svarer C, Frokjaer VG, Knudsen GM, Greve DN, Fisher PM. Functional connectivity of the dorsal and median raphe nuclei at rest. Neuroimage. 2015;116: 187–195.](http://paperpile.com/b/PfkOzA/916xP)

16. [Bär K-J, de la Cruz F, Schumann A, Koehler S, Sauer H, Critchley H, et al. Functional connectivity and network analysis of midbrain and brainstem nuclei. Neuroimage. 2016;134: 53–63.](http://paperpile.com/b/PfkOzA/k5CYJ)

17. [Amunts K, Kedo O, Kindler M, Pieperhoff P, Mohlberg H, Shah NJ, et al. Cytoarchitectonic mapping of the human amygdala, hippocampal region and entorhinal cortex: intersubject variability and probability maps. Anat Embryol . 2005;210: 343–352.](http://paperpile.com/b/PfkOzA/5Hr7w)

18. [Eickhoff SB, Stephan KE, Mohlberg H, Grefkes C, Fink GR, Amunts K, et al. A new SPM toolbox for combining probabilistic cytoarchitectonic maps and functional imaging data. Neuroimage. 2005;25: 1325–1335.](http://paperpile.com/b/PfkOzA/xeG15)

19. [Yeo BT, Krienen FM, Sepulcre J, Sabuncu MR, Lashkari D, Hollinshead M, et al. The organization of the human cerebral cortex estimated by intrinsic functional connectivity. J Neurophysiol. 2011;106: 1125–1165.](http://paperpile.com/b/PfkOzA/yYBlQ)

20. [Apkarian AV, Bushnell MC, Treede R-D, Zubieta J-K. Human brain mechanisms of pain perception and regulation in health and disease. Eur J Pain. 2005;9: 463–484.](http://paperpile.com/b/PfkOzA/qYs7z)

21. [Duerden EG, Albanese M-C. Localization of pain-related brain activation: A meta-analysis of neuroimaging data. Human Brain Mapping. 2013. pp. 109–149. doi:](http://paperpile.com/b/PfkOzA/J1ZPJ)[10.1002/hbm.21416](http://dx.doi.org/10.1002/hbm.21416)

22. [Wager TD, Atlas LY, Lindquist MA, Roy M, Woo C-W, Kross E. An fMRI-based neurologic signature of physical pain. N Engl J Med. 2013;368: 1388–1397.](http://paperpile.com/b/PfkOzA/V7IID)

23. [Yarkoni T, Poldrack R a., Nichols TE, Van Essen DC, Wager TD. Large-scale automated synthesis of human functional neuroimaging data. Nat Methods. 2011;8: 665–670.](http://paperpile.com/b/PfkOzA/AKPet)

24. [Snoek J, Larochelle H, Adams RRP. Practical Bayesian Optimization of Machine Learning Algorithms. Adv Neural Inf Process Syst. 2012; 1–9.](http://paperpile.com/b/PfkOzA/iKPy4)

25. [Jeffreys H. The Theory of Probability. OUP Oxford; 1998.](http://paperpile.com/b/PfkOzA/0qOTf)

26. [Carpenter B, Gelman A, Hoffman MD, Lee D, Goodrich B, Betancourt M, et al. Stan: A probabilistic programming language. J Stat Softw. 2017;76. Available:](http://paperpile.com/b/PfkOzA/AyMr0) <https://www.osti.gov/biblio/1430202>

27. [Goodrich B, Gabry J, Ali I, Brilleman S. rstanarm: Bayesian applied regression modeling via Stan. rstanarm: Bayesian applied regression modeling via Stan. 2018.](http://paperpile.com/b/PfkOzA/QSGy8)

28. [Rouder JN, Speckman PL, Sun D, Morey RD, Iverson G. Bayesian t tests for accepting and rejecting the null hypothesis. Psychon Bull Rev. 2009;16: 225–237.](http://paperpile.com/b/PfkOzA/GFkYc)

29. [Gronau QF, Singmann H, Wagenmakers E-J. bridgesampling: An R Package for Estimating Normalizing Constants. 2017. doi:](http://paperpile.com/b/PfkOzA/9XKMG)[none](http://dx.doi.org/none)

30. [Bates D, Mächler M, Bolker BM, Walker SC. Fitting linear mixed-effects models using lme4. J Stat Softw. 2015;67: 1–48.](http://paperpile.com/b/PfkOzA/hIMza)

31. [Morey RD, Rounder J. BayesFactor: An R package for computing Bayes factors in common research designs. Retrieved; 2012.](http://paperpile.com/b/PfkOzA/Y3el9)

32. [MacKinnon DP, Lockwood CM, Williams J. Confidence Limits for the Indirect Effect: Distribution of the Product and Resampling Methods. Multivariate Behav Res. 2004;39: 99–128.](http://paperpile.com/b/PfkOzA/rylYv)

33. [Wilkinson GN, Rogers CE. Symbolic Description of Factorial Models for Analysis of Variance. Appl Stat. 1973;22: 392.](http://paperpile.com/b/PfkOzA/lVWIU)
