# Supplementary figures and images for: A multistudy analysis reveals that evoked pain intensity representation is distributed across brain systems
Source: PLoS Biol. 2022 May 2;20(5):e3001620. doi: 10.1371/journal.pbio.3001620 (PMC9098029; doi:10.1371/journal.pbio.3001620)

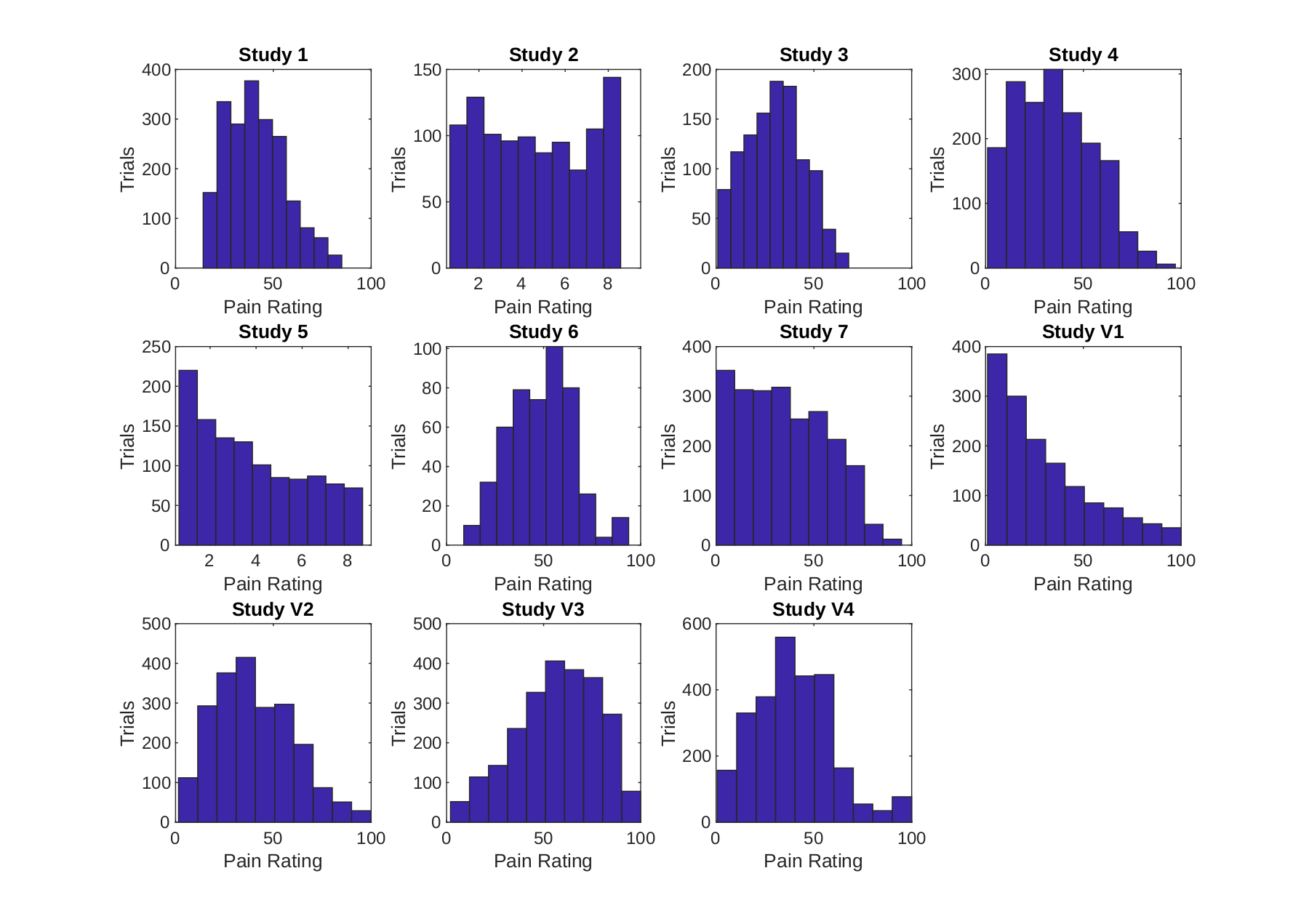

Supplement: S1 Fig — Data shown are unstandardized, and, instead, the x-axis was scaled to match the rating scale range offered to participants. Underlying data: https://github.com/canlab/petre_scope_of_pain_representation/tree/main/figureS1. (TIF) [file pbio.3001620.s003.tif]

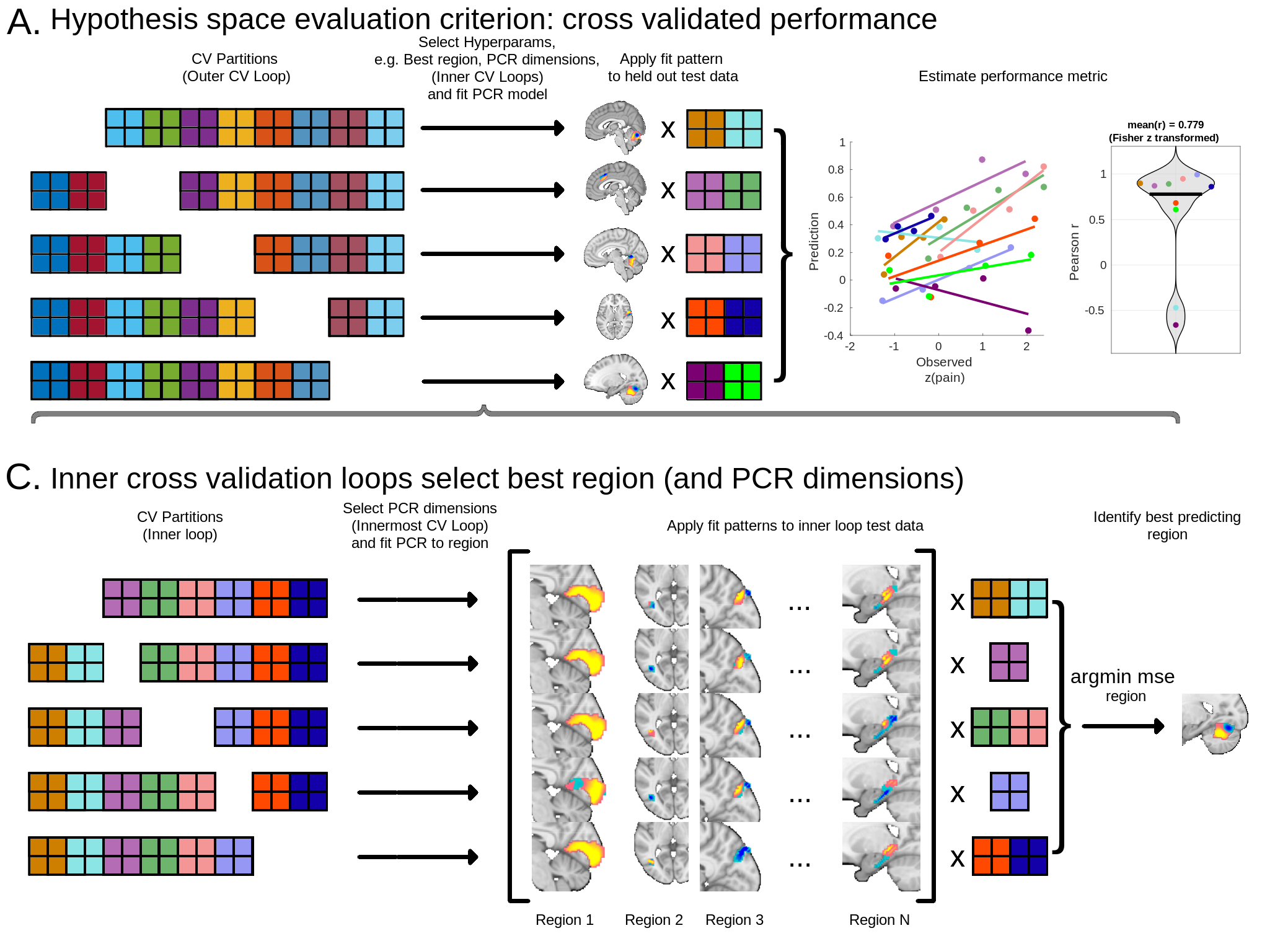

Supplement: S2 Fig — (A) We used PCR to learn a model from an area. We estimated model performance using 5-fold CV, training models on data averaged within pain intensity quartiles (smallest squares) for each participant (4× squares and one color per participant). Participant data were not fragmented across folds (shown), and studies were balanced across folds (not shown). We estimated model performance using within participant Pearson correlation of predicted and observed ratings. (B) The model fitting algorithm selected optimal model hyperparameters to minimize MSE, which it estimated using nested CV folds. In the case of multiarea hypotheses (A, top row), the algorithm treated region selection as a hyperparameter, and identified a single best region for each outer fold (folds illustrated in B). This region may have differed across outer CV folds. PCR dimensionality was optimized by estimating expected MSE in an additional innermost CV loop (3 levels of nested folds). In the case of distributed area hypotheses (A, bottom row), the algorithm only performed the latter step (2 levels of nested folds). PCR hyperparameter optimization folds are not illustrated. CV, cross-validation; MSE, mean squared error; PCR, principal component regression. (TIF) [file pbio.3001620.s004.tif]

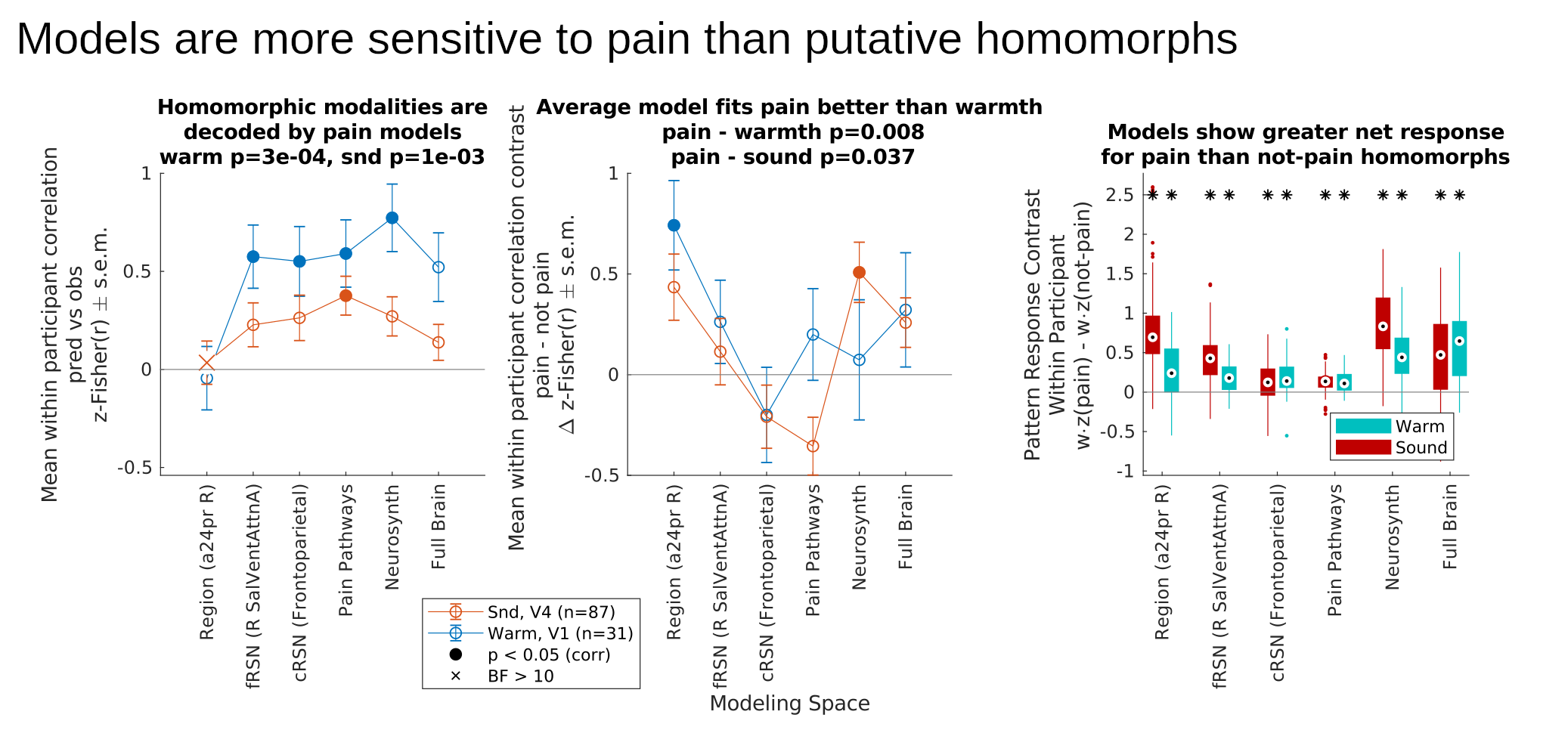

Supplement: S3 Fig — In validation studies where participants rated aversiveness of sound or warmth of nonpainful noxious heat as well as heat pain, models on average predicted nonpain ratings (left, warmth prediction p = 3e-4, sound prediction p = 0.001), and specifically fRSN, cRSN, and neurosynth models predicted warmth (p = 1.2e-3, 4.2e-3, and 9.8e-4 resp), while the pain pathways model predicted both warmth and sound ratings (p = 1.7e-3, p = 2.7e-4, resp). However, models predicted pain ratings better than not-pain ratings (center, pain > warmth, p = 0.008, pain > sound p = 0.037). Accuracy of pain rating predictions are indirectly indicated by net model response differences (right), which likewise showed greater responses for pain than not-pain stimuli in a manner roughly proportional to the intermodal within-participant differences in correlations (center). *Holm–Sidak α = 0.05 for 12 comparisons. Underlying data: https://github.com/canlab/petre_scope_of_pain_representation/tree/main/figureS3. cRSN, coarse resting-state network; fRSN, fine resting-state network. (TIF) [file pbio.3001620.s005.tif]
