# Supplementary material for: A multistudy analysis reveals that evoked pain intensity representation is distributed across brain systems
Source: PLoS Biol. 2022 May 2;20(5):e3001620. doi: 10.1371/journal.pbio.3001620 (PMC9098029; doi:10.1371/journal.pbio.3001620)
Supplement: Attachment — Submitted filename: responses to reviewers.pdf [file pbio.3001620.s012.pdf]

Responses to Reviews

We would like to thank the editor and reviewers for their efforts and thoughtful comments in assessing this manuscript. Based on these comments we have made substantial efforts to clarify the language, simplify the figures, and better organize the text to make our study accessible to a wide readership. The revisions are substantial, but in brief, we have undertaken the following major changes: (1) we shortened the introduction by 15%. (2) We updated the results text by moving a specificity analysis to the supplemental materials to accompany its corresponding supplementary figure. This analysis was a preliminary step that potentially distracted from our main specificity analysis, which now features more prominently in the main text. We restructured the remaining results sections in a modular manner, separating out components that had previously been interleaved, and elaborated on the motivating logic underlying several of these modules. We believe this has resulted in a less dense text and more streamlined narrative. (3) We shortened the discussion by 25%. (4) We restructured the methods in the main text to better mirror the presentation of the results, tightened up our methods by moving certain technical details to the supplement and consolidating redundant content. This resulted in a 20% shortening of the Methods. (5) We removed some jargon, changing hypothesis space to ‘modeling space’ (these are the only updates to figures 2, 5 and 7) and mega-analysis to multi-study analysis. Additional details on these and other changes can be found in our point by point responses below.

Thank you, and we hope you find these changes constructive and effective at addressing your prior concerns.

COMMENTS FROM THE ACADEMIC EDITOR:
The authors make the assertion in the abstract 'These findings quantify the extent that representation of evoked pain experience is distributed across multiple cortical and subcortical systems'. But this is actually false, because it conflates the ability to predict experience with experience itself (i.e. it doesn't show that this representation is necessary or sufficient for experience). It should read 'the representation of evoked responses that can be used to predict pain experience', which is fundamentally different.

We agree that the suggested revision is a more accurate way to characterize our findings, and we have changed this sentence. Indeed, in our view,

“representation” is a widely used concept but one that is poorly operationally defined. One of the ways to operationally define a putative “representation” of a mental state or behavior is that it predicts that state in a sensitive and specific fashion. Ideally, direct manipulations of the brain substrate would be included to assess causality, though this is not possible here. We now more carefully describe our operational definition and refer to them as“formal representations that decode pain intensity. It now reads: “These findings quantify the extent to which multiple cortical and subcortical systems are needed for formal representations that decode pain intensity, especially heat pain. They suggest that the representations sufficient to decode pain experience may not be circumscribed by any elementary region or canonical network, and provide a blueprint for evaluating the spatial scale of information in other domains”.

Reviewer #1, Jian Kong: In this manuscript, the authors compared multivariate predictive models to investigate the spatial scale and location of acute pain intensity representation. This is a very interesting study; the analysis is appropriate, and the manuscript is well-written and organized. Please see my following comments, which hopefully can improve the manuscript.

Thank you for your positive comments, and critical feedback!

1. As the authors mentioned, distinct representations have been demonstrated for different types of pain including somatosensory and vicarious pain, and different kinds of clinical pain. It seems to the reviewer that this study has been focused on the experimental heat pain. I would suggest the authors change the title, abstract and other related sections to be clarify / emphasize this point.

We in general agree with the reviewer, with one reservation. We find that the models that are more predictive of heat pain in our study are also better at distinguishing multimodal pain (visceral, mechanical, and thermal) from putatively homomorphic tasks (i.e. that share similar task demands and may result in similar patterns of brain responses; Figure 8). This suggests that our findings are more general and are not limited only to heat pain. As a result, we have taken a middle ground between our current wording and the reviewer’s suggested wording. We have modified our abstract to make clear that our models are trained on heat pain, have included a comment regarding evidence that our results generalize to pain across modalities, while emphasizing that they apply ‘especially’ to heat pain, but have not added ‘heat’ in our study title. The abstract now reads “we compared multivariate predictive models to investigate the spatial scale and location of acute heat pain intensity

representation" and "These findings quantify the extent that multiple cortical and subcortical systems are needed for formal representations that decode pain intensity, especially heat pain".

2. Please specify the pain intensity rating ranges for each study. Can these models predict mild-moderate pain ratings better than intensive pain ratings or vice versa?

We have added a figure in the supplement showing the rating ranges for each study (Supplementary Figure S1). As we describe in more detail below, the models predict mild-moderate pain ratings and moderate-strong pain ratings equally well.

We tested for different sensitivity of our models to low vs. high intensity pain ratings. Specifically, we tested for a nonlinear relationship between predicted and observed ratings in each of our final multistudy models (figure 5a). Using Wilkinson notation, we tested the following model:

Pred ~ obs + obs^2 + 1 + (obs + obs^2 + 1 | subject) + (obs + obs^2 + 1 | study)

If there is a differential sensitivity to low pain vs. high pain ratings one would expect a nonlinear relationship between predicted and observed ratings that reflects this (i.e., larger slopes for high-intensity). Therefore we included a nonlinear function of observed pain, including random slopes for participants and studies. We tested this model on all six multi-study models (regional, fRSN, cRSN, pain pathways, neurosynth and full brain). We used Satterthwaite correction for degrees of freedom. All models show significant linear effects between predicted and observed pain, but no model showed a significant nonlinear effect ($p > 0.3$ in all cases). This suggests that the models are equi-sensitive to pain across the range of intensities. We additionally tested a simpler model that dropped random slope and nonlinearity effects at the subject level, but was otherwise the same as the above, and we obtained the same results. Thus, we found no evidence that our models predicted more intense pain ratings better or worse than less intense pain ratings.

3. If the reviewer is correct, a 0 - 10 VAS scale was used to evaluate the pain intensity in these studies. One question is if transforming continuous pain intensity rating into a category scale (for instance dividing the pain intensity into mild, moderate, and strong), will that change the results significantly? A related

question is do the authors believe that the brain codes the pain intensity as a continuous variable or a category variable in the real world?

Thank you for these interesting questions. The reviewer is correct that the rating scales are continuous. Collapsing these continuous variables to categories would remove significant variability from our observed values; however, our models would still yield continuous outcomes because they are linear regression models. The resultant model performance would be trivially reduced relative to what we currently show due to reduction in variance in the outcome variable due to binning, but not from the predicted score. A test for categorical pain outcomes would ideally use multinomial classification models rather than regression models, which we could in principle have implemented, but this would change most of our data analysis approach from start to finish, and to our knowledge has no precedent in the pain decoding literature for us to build on, further complicating implementation and presentation of our results.

Whether pain is coded categorically or continuously in the brain is certainly an interesting question, but outside the scope of our study, and possibly even outside the scope of our measurement methodology (BOLD fMRI). There is, however, both psychophysical and neurophysiological work suggesting that pain is a continuous signal. Recordings from nociceptive neurons, including wide dynamic range neurons, have shown linear rate coding [1–3]. While primary nociceptors have shown specific firing thresholds [4], the average over many such neurons with variable thresholds will yield a continuous rate-code for intensity (i.e. more neurons are recruited at higher intensity). Meanwhile, the psychophysics literature complements this data by showing that magnitude perception is continuous in general [5,6] and in pain specifically [3], indicating that signals remain continuously coded even after they are transformed from unconscious nociceptive signals into a conscious perception of pain.

There are nevertheless still open questions regarding how the brain encodes pain magnitude information, and whether there may be categorical differences in its response throughout the intensity range. Future studies are needed to address these issues. For the time being we believe the evidence tilts in favor of pain intensity being a continuous variable in the brain.

Reviewer #2: The manuscript by Petre and colleagues describes the use of machine learning for the decoding of pain intensity across different studies. This is an interesting but very long and complex manuscript. I see no major issue with this study but do not see any fundamental advance compared to the

previous machine learning application on the neurological pain signature from the authors. Such an incremental advance is usually regarded as insufficient for journals of the likes of Plos Biology.

Our work builds upon previous machine learning models of pain and provides new insights in several substantive ways. Although past work has demonstrated that whole-brain models tend to perform well when predicting pain reports, formal model comparisons across studies have been lacking. This raises several issues. First, it is possible that simple (single region) models *do* perform comparably to whole-brain models, but the relevant tests have not been consistently reported in past work; we do so here. Second, whole-brain models could perform well in limited experimental contexts (e.g., when predicting pain from one type of stimulus) but may not generally be associated with improved predictive performance. In the absence of formal comparisons across spatial scales, it is unclear which of these possibilities is most likely. We perform such formal comparisons, providing the most definitive evidence to date (to our knowledge) on whether pain-predictive representations are truly distributed across regions and systems. The methodology we develop could be used in other fields as well, to provide formal statistical inferences on the distributed nature of brain representations. We have made this point clearer in the first paragraph of our discussion:

"These results build on prior studies that examined only one particular spatial scale *a priori* (e.g. (Wager et al. 2013; Marquand et al. 2010) or investigated the spatial scope of pain-predictive representations under more restrictive conditions (Brodersen et al. 2012; Brown et al. 2011) by comprehensively quantifying information representation across scales and generalizing to heterogeneous evoked pain experiences across a diverse collection of task conditions, including mechanical and visceral pain. We thus demonstrate that evoked pain is represented preferentially by specific brain areas, but that multiple areas are required for accurate pain decoding."

Other novel features include: (1) we provide the first formal test in the neuroimaging literature (to our knowledge) of learner generalization, an important concept from machine learning. Learner generalization establishes whether a *class* of models, not just a single model, shows a property – here, whether predictive representations are distributed. (Please see below for more details). And (2) The most extensive characterization to date of which single regions best predict pain, including the anterior midcingulate cortex and posterior insula and secondary somatosensory cortex (area "OP1"). This is

important because many studies focus on characterizing representations and/or connectivity in individual brain areas and pathways, and our results provide valuable information on which regions show robust and generalizable predictions across studies, and precise localization of these areas and referencing to established atlases so the results can be built on in future studies.

Some recommendations:
The manuscript is very long, difficult to understand, and asks a lot from the average scientific reader in pain research. The introduction contains some redundant information, e.g. the first paragraph.
To shorten the manuscript I would suggest the authors focus on the essential aspects of the study. My suggestions
move analysis steps before ML to supplement as they are standard procedures.
Shrink introduction
A more focussed discussion.

Thank you for these suggestions. We have removed some of the redundancy, shortening the introduction word count by about 15%. We have focused the discussion, reducing the length by about 25%. We hope that this has made the paper clearer and easier to digest.

We balanced this shortening and simplification with the need to retain key methodological details in the main manuscript particularly those needed to address issues raised by other reviewers. For instance, we include key details about binning trials by pain quartiles, and removing overall activation magnitude by standardizing single-trial images, which has been a conceptual discussion point in past papers [7].

The results part is difficult to read. I understand that the manuscript structure of Plos Biol with a closing methods section requires a more extended results part. However, understanding the results without having read the methods section is not doable for this complex study anyway.

Thank you for pointing this out, and we have worked to make the text clearer. We have moved some minor results from the specificity analysis to the supplement (while retaining the most important specificity analysis in the main text), allowing us more room to elaborate on the methods and motivating logic in the main document. We have revised this section to make it less dense and more readable. There are three major changes in particular worth noting:

1. We have rewritten the sections describing figures 3 and 4, since this seemed to be a particular point of confusion for reviewer 3 as well. The section previously subtitled “MVPA model characterization” is now broken into two sections, “Many modules predict pain well” and “MVPA models are nested across studies,” allowing us to better clarify the significance of model interrelations.
2. We have additionally written more regarding the motivation behind learner generalization performance. Model generalization is about testing whether a specific model “works” on new datasets (or is better than another specific model), whereas learner generalization is about testing whether a *class* of model (e.g., single-region, multiple-network) is better than another class of models. The latter is more difficult to demonstrate, more rarely assessed and it is one of the novel contributions here.
3. We have elaborated on the mediation analysis and put it into its own subsection. We will discuss this further with respect to Reviewer #3’s comments regarding our mediation analysis.

The methods section would benefit from a bit more structure.

We have restructured the methods section to more closely follow the layout of the results. Our intention was to structure them such that as the reader progressively reads further into our results they also will find progressively later method subsections relevant. This way we hope for the methods to be more serially readable, so that the reader doesn’t need to jump back and forth between earlier and later parts while working through our results. We have also signposted the relevant methods section more frequently in the results. Finally, we have renamed some of the subsections in the results, methods and supplemental methods to mirror one another more closely, and moved some of the explanation of our motivating logic from the methods section into the results (especially some of the logic previously explained in the subsection “fMRI data analysis: Summary”; that section has now been removed because it is redundant).

I do not fully understand why it is not possible to use a leave-one-out approach for all 11 studies. In my view this can be done and is less prone to subjective division between training data and application data.

Leave-one-study-out cross-validation on all 11 studies would also have been a reasonable approach, though we believe the strategy we adopted here is more

rigorous. We appreciate the opportunity for further discussion and elaborate on our rationale below.

We used cross validation to estimate performance using 7 studies, and then tested performance in 4 held out studies to confirm the accuracy of the models' estimates. The reviewer suggests that instead of performing initial model evaluation in 7 studies, we instead train models on 10 studies and test on the 11th, repeating for every potential split. This replaces performance estimation using cross validation on 7 studies with cross-validation performed on 11 studies, but leaves us with no final hold out set to validate those estimates.

We agree that cross-validation across 11 studies would also be a reasonable approach in principle, but we elected to separate a test dataset (4 studies) and keep that as a separate test rather than including it in cross-validation for several reasons. First, cross validation is a biased estimation procedure, and the nature of the bias depends in a complex way on the data, hyperparameter selection, and cross validation scheme [8,9]. Second, validity of cross validation estimates depends on independence of training and test data. However, in the course of algorithm design and implementation it is possible that the statistical properties of the dataset may unconsciously influence the analyst's implementation choices. This risks introducing a certain circularity where a test fold's model is in part chosen based on properties of the test data gleaned during prior analyses (e.g. code development and data exploration). Retaining a blind hold out set (our 4 studies in figure 7 here), guards against both forms of error by either confirming the validity of cross validation estimates or providing a warning of inferential errors. This approach is common in machine learning (e.g. [10]) and sees some applications in neuroimaging, e.g. [9]. As we had enough data to provide a good estimate of generalization in the test data set across several studies, we prioritized this over cross-validated accuracy estimates.

Another, final consideration to note is that we selected the training and validation datasets prior to analysis, so that the final models (only) were tested on the validation dataset. This prevents overfitting and optimistic biases. Changing the holdout scheme at this point could compromise the rigor of the approach, as we would not have a perfectly clean application to validation datasets that have not been touched during the model development process.

I would strongly recommend the authors to drop MEGA. The number of included studies and participants is nowhere near MEGA. It just sounds [...]. The senior

author may consider that this terminology would be mostly bound to the first author, which could be embarrassing for him in his future scientific career (I know what I'm talking about).

We were also uncertain about this term, and have dropped “mega” as suggested. Several groups have used it to describe participant-level analyses across multiple studies (which was our intention here), but it is not in widespread use. We did not intend it as a comment on the size of the dataset, merely on the approach we chose. Based on the comments of reviewer 2 we have removed this term and now simply refer to multi-study analyses instead of “mega-analysis”.

More details on this decision are below.

A review of the literature on mega-analysis and meta-analyses of individual participant data (an equivalent term) reveals conflicting usages over time. We identified a selection of recent neuroimaging and cognitive science studies that use these terms. They had a variety of sample sizes spanning roughly an order of magnitude from 3 datasets to 42 datasets and from <100 subjects to >3000 [11–15]. However, when examining historical usage of the label we found that it originated with studies in the medical literature which made systematic efforts to pool comprehensive datasets from a large collaboration of research groups [16–18], for instance to pool results from multiple clinical trials. Our study was not designed for this purpose (i.e., it was not intended to be a systematic meta-analysis). Therefore, in summary, given the conflicting uses of the term it seems clear now that our use of ‘mega-analysis’ may cause some confusion. We have consequently dropped it in this revision.

I wonder if any of the co-authors would understand the study in full detail. I do not feel competent enough to evaluate the methods due to its complexity. I have no idea how the analyses in the supplement relate to the analyses in the main document. I guess there are more pain researchers who would find this manuscript incomprehensive and unreadable.

We have made efforts to simplify the analysis, and moved some of the technical details to the supplement to streamline the main document.

In terms of the question about “how the analyses in the supplement relate to the main document”, the previous version of the supplementary materials didn’t actually include any supplemental results. However, we have revisited all

sections of the supplement and attempted to clarify their relation to the main text. We provide more detail on this below.

Figure S2 shows that tests for specificity produce the same results whether models are applied to single trial BOLD contrast maps, or to contrast maps averaged across trials (as in a traditional GLM analysis). In our prior version of the manuscript this figure was explicitly referenced and discussed at the start of the main text results subsection titled 'Models capture pain specific representations.' In the interest of streamlining the results section and addressing potential confusion relating this text to figure S2 we have made the following changes: (1) we moved the text associated with figure S2 into the supplemental results, so that it's in all in one document. (2) We have elaborated on the motivation of this analysis at its new location in the supplement and clarified its relationship to the main document. We do this twice, once at the start of the supplemental results section, and again at the end.

We have additionally included and cross-referenced tables S1-S4. These are numerical versions of results illustrated visually in the main document (e.g. the actual numerical values underlying the bar graphs of figure 5 are provided by table S3), are sign posted in the main document, and the associated main document figures are sign posted again in the table legends in the supplement. If these were the analyses the reviewer happened to be unclear about, we're confident things will become clear upon a second look.

Finally it's possible the analyses described in the supplemental methods were not clear. We have signposted Figure 4 in the bootstrap test methods description, to indicate that these methods pertain to the results in that figure, signposted the mediation analysis to Figure 6 as well, and sign posted the Bayesian hypothesis test to Figures 5, 7 and 8 where null hypothesis tests are illustrated.

Finally, we note that much of the difficulty in our manuscript derives from the fact that we're importing methods from the machine learning literature and statistical learning theory, which is inherently challenging, and trying to bring it to a broader audience. Readers are in particular likely to have to make an effort to understand the distinction between model and learner/algorithm generalization performance, for instance. It was new to us too, but given the increasing importance of machine learning algorithms to neuroscience research and society more broadly, we think it is crucial to expand the scope of our understanding from models to learning processes, despite the complexity of

this move. That said, we have made efforts to clarify some of these more nuanced points in the text. In particular we have rewritten the results subsection “In a general sense, distributed models will do best: Learner generalization performance” and focused the discussion more on this issue.

Notably, a similar concern might be raised against the use of Bayes factors, which likewise are difficult to implement, and their use is beyond the current expertise of many pain researchers (see, e.g., the Bayesian hypothesis tests section of our supplement, which elaborates on a number of approaches that have been recently developed, and that are novel to our field, and note the relative lack of null hypothesis tests in cognitive science more generally). Nevertheless, these methods enjoy widespread advocacy in cognitive science and are generally celebrated by readers. We hope our work on inference over learning processes will be similarly received.

Reviewer #3: The article from Petre and colleagues presents a novel mega-analysis of fMRI studies using multivariate pattern analysis methods to consider the performance of different models - each with its own interesting anatomical relevance. The methods are novel and permit an interesting window into scale of pain processing in the brain using high quality data with enhanced sensitivity offered by the mega-analysis. The analysis of specificity with non-noxious data, and the external validation results, lead me to be encouraged and convinced by the approach. However, I have a few comments and queries for the authors about aspects of the manuscript, specifically some decisions on interpretation and methods employed.

1. In the abstract, and (to a lesser degree introduction) the authors imply that the scale of representation of pain is controversial. I am not sure about this. Possibly it is [my] own bias, but I believe most researchers are convinced by the fact that pain is present across cortical-subcortical systems. Perhaps the controversy referred to comes from recent papers that argue for a salience explanation, but it reads a little as though there is a genuine debate with a body of scientists arguing for localised modular centres of pain processing. Whilst this may have been true historically, I don't think it is the case now and would prefer to present this work as an interesting and valid comparison of models, rather than the resolution of an ongoing debate.

We have taken this suggestion and now present the issue in terms of integration and reconciliation of complementary perspectives rather than in terms of a ‘debate’. This builds off the historical theme we introduced in the first paragraph of the introduction, and is consistent with the credit we give to modular perspectives in the discussion.

2. The decision making for organising the data into quartiles is not very clear to me. This is important because it can clearly affect the results, but it comes to us as a fairly arbitrary decision. I see the computational explanation, but why not tertiles or 5ths which would be similarly feasible? It would be good for the reader to understand the decision making here.

We have added some comments to the methods regarding this choice, and specifically why tertiles are not optimal. In the “behavioral analysis” methods subsection we now write, “Quartiled data in particular was selected rather than some other quantile interval (e.g. tertiles or quintiles) because it has been established as a convention by previous studies [19] since it is the minimum level needed for all measures to contribute to estimates of linear intensity effects. With tertiles, for instance, the middle quantile only affects an intercept estimate, while the intensity effect is fully determined by the lowest and highest quantiles.”

That said, we do not believe our choice to average over multiple single trial events is a critical one for the validity or scientific utility of the present findings. Most neuroimaging research is premised on the fact that responses averaged over stimulus events adequately represent the constituent stimulus events. As a result, traditional GLM analyses typically represent all presentations of a single stimulus category with a single regressor, producing a single map, which is mathematically equivalent to averaging over the single trial responses of the constituent elements. Thus our approach mirrors standard data analysis strategies in the field, except that we represent pain quartiles as ‘single categories’ in this sense. Our only concern was to provide our models with adequate degrees of freedom to model the response outcome, and reduce the computational and memory demands of model training. In past work we have compared prediction using quartiles and single-trial data (no averaging), for example, and found very similar results. There are some variations in the sources of variance the models are sensitive to, but these do not bear substantively on our hypotheses here. The single-trial models are, however, orders of magnitude more costly (in computation time and memory) to train.

3. The value of some mediation analyses is not immediately clear to me. The first mediation we are presented with seems like a reasonable attempt to understand how the brain models capture experimental manipulation in the data. I see that this can enhance interpretation, but why utilise only the full brain

model? I can see some argument for using the other distributed models which according to some measures outperformed full-brain, or perhaps using mediation to tease apart differences in performance of different models. To inly use the full model is strange to me.

We focus on the full brain model because it is the most comprehensive in the sense that it is correlated with models at other scales (i.e., other models include subsets of features with similar weights), and it alone has sufficient spatial scope to guarantee overlap with all variations of the others (e.g. all modules which might have been selected across CV folds). Because of these properties, and because of the historical focus on whole-brain prediction we chose it *a priori* for more targeted testing (see also brain vs. region comparisons in Figure 5, for instance). We explain this rationale more clearly now.

We had in fact performed mediation analyses for all models. However we did not include these additional mediation analyses in the previous version of the manuscript because we felt there was already a lot to absorb and inclusion of these additional analyses seemed likely to be lost among the details. Perhaps, however, their absence may in fact distract some readers. Given the reviewer's questions, we've reconsidered our choice and now include tables with mediation results for other models in the supplemental materials (Table S5 and S6).

Tables S5 and S6 show that models at other scales, especially multisystem models, also mediate multi-dimensional representations of pain report. This is consistent with the conclusion we draw in the discussion that multisystem and full brain models subsume and build upon smaller scale models (also supported by the pattern similarity analysis earlier in the results section). It also helps us understand how these models might show some residual response to putatively homomorphic tasks, since these tasks likely overlap on some dimensions with pain tasks, and our models respond to a multidimensional pain representation. This is a point we touch on in the discussion.

4. To follow on from the previous point, I found the mediations in the learner generalized performance section to be problematic. Firstly, it required some pooling of data across studies which are quite different, a problem that the authors mention (because it limits their interpretation) at the end of this section themselves. In a paper that is quite dense, I am not convinced of the benefit or need for these analysis.

We have reorganized and partially rewritten some of the mediation analysis to better convince readers of the benefit of these analyses. They are now separated into their own subsection. In particular, it was important to us to show that brain models accurately represent our experimental conditions. The mediation analysis provides a connection from the brain models to the behavioral response at the beginning of our results section, and ties them together. Although we do not have space to elaborate on this at length in the discussion, one of the premises of our study, referenced in the introduction, is that prior efforts to study the scope of pain representation have not 'generalized across diverse task conditions.' The mediation analyses serve as a way of improving on this record. That is the need and benefit for these analyses.

As for the need to pool data across studies, this may not be as problematic as the reviewer at first assumes. Bootstrap tests of small samples suffer from a known lack of power, a fact we now clarify in our results section, and we provide a citation which elaborates extensively on this fact in the specific context of bias corrected bootstrap confidence interval estimation in mediation analysis. Because we are bootstrapping subjects, our samples are in fact small when evaluated one study at a time (16-30 subjects/randomization blocks) relative to the sample sizes recommended by published simulation studies in the statistical literature (>100) (See [20] for details). However, when pooling across studies we have 171 subjects, which is above the recommended sample size. Additionally, for this particular analysis in question we are not even interested in individual models, but rather in learning processes (i.e., learner generalization). These learning processes are always evaluated by pooling data across studies (e.g. figure 5b). Doing so again for the mediation analysis is in principle no different, as long as the reader understands that the results reflect what can be learned by a class of models rather than what has been learned in any particular instance. We would ideally be able to demonstrate that multiple dimensions of pain are mediated simultaneously by individual models, but the evidence is highly suggestive of this fact, even absent statistical power to make the point formally. We make greater efforts to clarify this in the revision.

5. I realise this is quite a long paper, but I really feel that a very brief explanation of the anatomical underpinnings of the models (particularly those with anatomical relevance such as PP and RSN regions) should be mentioned in the text of the article, rather than just supplemental. The detail can still be signposted in supplemental.

We have updated the methods section with further descriptions of the anatomical underpinnings of our PP and RSN (see Defining model spaces and inputs, and in particular where we write, “Both fRSN and cRSN parcellations were derived from clustering of brain areas based on their functional connectivity profiles, but differ in their clustering threshold. Illustrations (figure 2) show that some networks primarily involve adjacent areas while others involve several disconnected areas of the cortex.” and also later write in regards to PP that “These regions include thalamic and brainstem nuclei, amygdala, insular, somatosensory and mid cingulate cortex.”).

6. On Figure 4, I found the text around wheel plot figures to be quite difficult, and the grey lines that came off some labels to lead to the brain maps were also a little problematic. It is a minor thing but I think these bits could be a little clearer.

We have removed the wheel plots. The figure was busy, and removal of those plots helps focus attention on the main results that are actually discussed by the main text. We have made additional modifications to the bootstrap maps that were associated with the wheel plots (we conjoined the maps) to better connect them to the pattern similarity analysis illustrated by the correlation matrix as well.

Reviewer #4: This is an excellent mega-analysis investigating whether pain representation is best characterized as localized, multi brain system, or whole brain. The authors provide a detailed and transparent description of their approach and results from this complex analysis. The results will have an impact on future planning of studies examining the representation of pain in the brain. Great contribution and wonderful and explanatory figures.

Thank you, much appreciated!

Bibliography

1. Andrew D, Craig AD. Responses of spinothalamic lamina I neurons to maintained noxious mechanical stimulation in the cat. J Neurophysiol. 2002;87: 1889–1901.

2. Price DD, Dubner R. Neurons that subserve the sensory-discriminative aspects of pain. Pain. 1977;3: 307–338.

3. Price DD. Psychological and Neural Mechanisms of Pain. Raven Press; 1988.

4. Treede RD, Meyer RA, Raja SN, Campbell JN. Evidence for two different heat transduction

mechanisms in nociceptive primary afferents innervating monkey skin. J Physiol. 1995;483 (Pt 3: 747–758.

5. Stevens SS. To Honor Fechner and Repeal His Law: A power function, not a log function, describes the operating characteristic of a sensory system. Science. 1961;133: 80–86.

6. Stevens SS. Psychophysics: Introduction to Its Perceptual, Neural and Social Prospects. Routledge; 2017.

7. Hu L, Iannetti GD. Painful Issues in Pain Prediction. Trends Neurosci. 2016;39: 212–220.

8. Jiang G, Wang W. Error estimation based on variance analysis of k -fold cross-validation. 2017;69: 94–106.

9. Varoquaux G, Raamana PR, Engemann DA, Hoyos-Idrobo A, Schwartz Y, Thirion B. Assessing and tuning brain decoders: Cross-validation, caveats, and guidelines. Neuroimage. 2017;145: 166–179.

10. Xu Y, Goodacre R. On Splitting Training and Validation Set: A Comparative Study of Cross-Validation, Bootstrap and Systematic Sampling for Estimating the Generalization Performance of Supervised Learning. J Anal Test. 2018;2: 249–262.

11. Haugg A, Renz FM, Nicholson AA, Lor C, Götzendorfer SJ, Sladky R, et al. Predictors of real-time fMRI neurofeedback performance and improvement--A machine learning mega-analysis. Neuroimage. 2021; 118207.

12. Visser RM, Bathelt J, Scholte HS, Kindt M. Robust BOLD responses to faces but not to conditioned threat: challenging the amygdala's reputation in human fear and extinction learning. J Neurosci. 2021. doi:10.1523/JNEUROSCI.0857-21.2021

13. Merritt K, McGuire PK, Egerton A, Aleman A, Block W, Bloemen OJN, et al. Association of age, antipsychotic medication, and symptom severity in schizophrenia with proton magnetic resonance spectroscopy brain glutamate level: a Mega-analysis of individual participant-Level data. JAMA Psychiatry. 2021;78: 667–681.

14. Jalbrzikowski M, Hayes RA, Wood SJ, Nordholm D, Zhou JH, Fusar-Poli P, et al. Association of Structural Magnetic Resonance Imaging Measures With Psychosis Onset in Individuals at Clinical High Risk for Developing Psychosis: An ENIGMA Working Group Mega-analysis. JAMA Psychiatry. 2021. Available: https://jamanetwork.com/journals/jamapsychiatry/article-abstract/2779599

15. Bigdely-Shamlo N, Touryan J, Ojeda A, Kothe C, Mullen T, Robbins K. Automated EEG mega-analysis I: Spectral and amplitude characteristics across studies. Neuroimage. 2020;207: 116361.

16. Stewart LA, Clarke MJ. Practical methodology of meta-analyses (overviews) using updated individual patient data. Cochrane Working Group. Stat Med. 1995;14: 2057–2079.

17. Chalmers I. The Cochrane collaboration: preparing, maintaining, and disseminating systematic reviews of the effects of health care. Ann N Y Acad Sci. 1993;703: 156–63; discussion 163–5.

18. Tierney JF, Vale C, Riley R, Smith CT, Stewart L, Clarke M, et al. Individual Participant Data

(IPD) Meta-analyses of Randomised Controlled Trials: Guidance on Their Use. PLoS Med. 2015;12: e1001855.

19. Wager TD, Atlas LY, Lindquist MA, Roy M, Woo C-W, Kross E. An fMRI-based neurologic signature of physical pain. N Engl J Med. 2013;368: 1388–1397.

20. MacKinnon DP, Lockwood CM, Williams J. Confidence Limits for the Indirect Effect: Distribution of the Product and Resampling Methods. Multivariate Behav Res. 2004;39: 99–128.
