# Supplementary material for: A multistudy analysis reveals that evoked pain intensity representation is distributed across brain systems
Source: PLoS Biol. 2022 May 2;20(5):e3001620. doi: 10.1371/journal.pbio.3001620 (PMC9098029; doi:10.1371/journal.pbio.3001620)
Supplement: Attachment — Submitted filename: response_to_reviewers.pdf [file pbio.3001620.s013.pdf]

Dear Editors,

Please find enclosed the final editorial comments and our responses indicating changes made to address the editorial requirements. Thank you for your careful and thoughtful handling of our manuscript - we appreciate it!

Thank you for submitting your revised Research Article entitled "Evoked pain intensity representation is distributed across brain systems: A Multistudy Analysis" for publication in PLOS Biology. I'm handling your paper for the final stages, as my colleague Luke Smith is currently on parental leave. I've now obtained advice from the original reviewers and have discussed their comments with the Academic Editor.

Based on the reviews, we will probably accept this manuscript for publication, provided you satisfactorily address the following data and other policy-related requests.

a) Please flip the title around to give us an active verb and avoid punctuation: "A multistudy analysis reveals that evoked pain intensity representation is distributed across brain systems"

We've made this change.

b) Please provide a blurb according to the instructions in the submission form.

We've made this change.

c) As this is a meta-analysis, we draw your attention to our relevant guidelines here: https://nam12.safelinks.protection.outlook.com/?url=https%3A%2F%2Fjournals.plos.org%2Fplosbiology%2Fs%2Fbest-practices-in-research-reporting%23loc-reporting-guidelines-for-specific-study-types&data=04%7C01%7CTor.D.Wager%40dartmouth.edu%7Cfc5d1c0c996a48181c8108da05c582dd%7C995b093648d640e5a31ebf689ec9446f%7C0%7C0%7C637828643111485335%7CUnknown%7CTWFpbGZsb3d8eyJWIjoiMC4wLjAwMDAiLCJQIjoiV2luMzIiLCJBTiI6Ik1haWwiLCJXVCI6Mn0%3D%7C3000&sdata=6MdChtCpMP2%2FI%2BdQ3dAnFAtGGdjaa2DNsG7cG0YYL8E%3D&reserved=0 Reports of systematic reviews and meta-analyses must adhere to the PRISMA statement as a guide, and include a completed PRISMA checklist and flow diagram to accompany the main text. Authors must also state within their Methods section whether a protocol exists for their systematic review, and if so, provide a copy of the protocol as Supporting Information.

We have addressed this point in correspondence with Dr Roland Roberts. Briefly, it was not our intention that this study be a systematic review or meta-analysis, and a prior reviewer also objected to such characterization. Dr Roberts agrees, and has indicated that we may disregard this request. We have included a copy of this correspondence at the end of this document.

d) Please address my Data Policy requests below; specifically, we need you to supply the numerical values underlying Figs 1B, 2, 3, 4, 5AB, 6, 7AB, 8, S1, S2AB, S3. Please also cite the location of the data clearly in each relevant main and supplementary Fig legend, e.g. “Data underlying this Figure can be found in S1 Data”.

We have provided underlying data as csv and nifti files available on github, and linked to these in the relevant figure legends. A few notes regarding particular figures.

Figure 5: In the process of obtaining numerical values we noticed a mistake in the figure: previously the estimated main effects degrees of freedom rather than F value were listed in Figure 7A (11.12 instead of 4.25). We have corrected this. Other values, including p-values, were correct as they were in 7A. For figure 7B we have corrected the main effects degrees of freedom estimated from 8.21 to 8.19, and p-value from 0.13 to 0.12. It appears these were legacy values carried over from an older version of the figure. The F-stat and conclusions were unaffected within the precision shown.

Figure 6: We have additionally made a correction to this figure. Previously we indicated some statistically significant values with a cross, however one statistically significant value was missing a cross. The cross superscript has been added. Additionally, the scatterplot was incorrect and has been updated. Previously the subsample of subjects shown did not correspond to those of the mediation analysis. Now they do. No statistics or quantitative conclusions were drawn from this scatterplot, and qualitatively the plot is indistinguishable.

Figure S2: This figure is a conceptual figure. It is meant to characiature an analytic pipeline, not convey data. There is no relevant underlying data to provide.

Figure S3: We could not obtain the exact data used to generate the figure in our last submission. This figure includes data from a study of warmth perception which was averaged within quartiles of warmth intensity ratings. Quartiling of some subjects’ warmth ratings is stochastic due to multiple ratings with the exact same value near quartile boundaries, which leads to an ambiguous

quartile assignment for those warmth trials. As a result we have generated a new figure and saved the data used to generate it in the process. We now offer this new figure as a substitute along with the data corresponding to it. Although some of the values in the plot have been altered subtly, none of the conclusions of our frequentist or bayesian tests were altered in this substitution. This issue is unique to warmth ratings, and these were not used elsewhere in our manuscript.

As you address these items, please take this last chance to review your reference list to ensure that it is complete and correct. If you have cited papers that have been retracted, please include the rationale for doing so in the manuscript text, or remove these references and replace them with relevant current references. Any changes to the reference list should be mentioned in the cover letter that accompanies your revised manuscript.

We expect to receive your revised manuscript within two weeks.

To submit your revision, please go to https://nam12.safelinks.protection.outlook.com/?url=https%3A%2F%2Fwww.editorialmanager.com%2Fpbiology%2F&data=04%7C01%7CTor.D.Wager%40dartmouth.edu%7Cfc5d1c0c996a48181c8108da05c582dd%7C995b093648d640e5a31ebf689ec9446f%7C0%7C0%7C637828643111485335%7CUnknown%7CTWFpbGZsb3d8eyJWIjoiMC4wLjAwMDAiLCJQIjoiV2luMzIiLCJBTiI6Ik1haWwiLCJXVCI6Mn0%3D%7C3000&sdata=NzP%2FYJuAY9KOPuOeiHF2UEa5iChbxpd7I%2FaYz%2FBGFV0%3D&reserved=0 and log in as an Author. Click the link labelled 'Submissions Needing Revision' to find your submission record. Your revised submission must include the following:
- a cover letter that should detail your responses to any editorial requests, if applicable, and whether changes have been made to the reference list
- a Response to Reviewers file that provides a detailed response to the reviewers' comments (if applicable)
- a track-changes file indicating any changes that you have made to the manuscript.

NOTE: If Supporting Information files are included with your article, note that these are not copyedited and will be published as they are submitted. Please ensure that these files are legible and of high quality (at least 300 dpi) in an easily accessible file format. For this reason, please be aware that any references listed in an SI file will not be indexed. For more information, see our Supporting Information guidelines: https://nam12.safelinks.protection.outlook.com/?url=https%3A%2F%2Fjournals.plos.org%2Fplosbiology%2Fs%2Fsupporting-information&data=04%7C01%7CTor.D.Wager%40dartmouth.edu%7Cfc5d1c0c996a48181c8108da05c582dd%7C995b093648d

640e5a31ebf689ec9446f%7C0%7C0%7C637828643111485335%7CUnknown%7CTWFpbGZsb3d8eyJWIjoiMC4wLjAwMDAiLCJQIjoiV2luMzIiLCJBTiI6Ik1haWwiLCJXVCI6Mn0%3D%7C3000&sdata=%2Bh6E1GFhSyHEeWZr9geKVSyPc52%2F6ni6udbs2Qk9V8c%3D&reserved=0

Published Peer Review History
Please note that you may have the opportunity to make the peer review history publicly available. The record will include editor decision letters (with reviews) and your responses to reviewer comments. If eligible, we will contact you to opt in or out. Please see here for more details:
https://nam12.safelinks.protection.outlook.com/?url=https%3A%2F%2Fblogs.plos.org%2Fplos%2F2019%2F05%2Fplos-journals-now-open-for-published-peer-review%2F&data=04%7C01%7CTor.D.Wager%40dartmouth.edu%7Cfc5d1c0c996a48181c8108da05c582dd%7C995b093648d640e5a31ebf689ec9446f%7C0%7C0%7C637828643111485335%7CUnknown%7CTWFpbGZsb3d8eyJWIjoiMC4wLjAwMDAiLCJQIjoiV2luMzIiLCJBTiI6Ik1haWwiLCJXVCI6Mn0%3D%7C3000&sdata=G57P5%2BLyF58d76tyCr3ah2RZGghLDsbrjjBXCLwQ5kQ%3D&reserved=0

Press
Should you, your institution's press office or the journal office choose to press release your paper, please ensure you have opted out of Early Article Posting on the submission form. We ask that you notify us as soon as possible if you or your institution is planning to press release the article.

Protocols deposition
To enhance the reproducibility of your results, we recommend that if applicable you deposit your laboratory protocols in protocols.io, where a protocol can be assigned its own identifier (DOI) such that it can be cited independently in the future. Additionally, PLOS ONE offers an option for publishing peer-reviewed Lab Protocol articles, which describe protocols hosted on protocols.io. Read more information on sharing protocols at
https://nam12.safelinks.protection.outlook.com/?url=https%3A%2F%2Fplos.org%2Fprotocols%3Futm_medium%3Deditorial-email%26utm_source%3Dauthorletters%26utm_campaign%3Dprotocols&data=04%7C01%7CTor.D.Wager%40dartmouth.edu%7Cfc5d1c0c996a48181c8108da05c582dd%7C995b093648d640e5a31ebf689ec9446f%7C0%7C0%7C637828643111485335%7CUnknown%7CTWFpbGZsb3d8eyJWIjoiMC4wLjAwMDAiLCJQIjoiV2luMzIiLCJBTiI6Ik1haWwiLCJXVCI6Mn0%3D%7C3000&sdata=tlL7OtKO0NUINTkQCNAdN6cfB7HqEra9%2FbJ4COXm1cc%3D&reserved=0

Please do not hesitate to contact me should you have any questions.

Best wishes,

Roli

Correspondence regarding systematic reviews and meta-analyses:

Dear Roli,

Thanks very much for the positive feedback. We're delighted that the reviewers found the manuscript improved and suitable for publication in PLoS Biology.

We're working on the final changes and journal format requirements now. However, we do have one question regarding point (c) below, and we would appreciate some guidance on this.

We described the paper as a "multi-study analysis" in the title because the intent of the study was not to perform a systematic survey of the literature, as is typical of systematic reviews and meta-analyses, but to perform an analysis on a specialized type of data — models of fMRI responses on single-trials, and their associations with pain and stimulus intensity — using available datasets from our laboratory. To our knowledge, single-trial data of noxious stimuli is not generally available aside from data we collected directly or that was prepared by our collaborators. We additionally note that in the initial review reviewer 2 commented that this study did not qualify as a "mega-analysis" (which is short for "meta-analysis of individual participant data"), and that we should not refer to it as such. We agreed with this, which motivated the characterization of the study as a "multi-study" analysis rather than a meta-analysis. Thus, it might be misleading to label the study a meta-analysis and include a PRISMA diagram.

We would appreciate your guidance on this point. We would be happy to include a modified PRISMA diagram if you feel that we should, but it may not be advantageous given the goals of the study.

Thank you for your advice, and for considering the study for publication in PLoS Biology. Also, all the best to Luke on his paternity leave.

Best regards,
Tor

Dear Tor,
Thanks for your email, and don’t worry about the autocorrect on my name – it’s been autocorrected to much worse in the past! I totally take your point regarding PRISMA – I think this request was a hangover from the previous description of your study, which tends to trigger us to include this request (to err on the safe side). But I agree with you that this would be unhelpful and potentially misleading in this case – please disregard this request.
I’ll pass on your regards to Luke – he and his wife now have a lovely baby daughter, and he should be returning to work in another month or so.
Best wishes and congrats on the nice paper!
Roli
